# Predictors of Radiographic and Symptomatic Hemorrhagic Conversion Following Endovascular Thrombectomy for Acute Ischemic Stroke Due to Large Vessel Occlusion

**DOI:** 10.7759/cureus.24449

**Published:** 2022-04-24

**Authors:** Kainaat Javed, Andre Boyke, Ishan Naidu, Jessica Ryvlin, Rose Fluss, Adisson N Fortunel, Joseph Dardick, Devikarani Kadaba, David J Altschul, Neil Haranhalli

**Affiliations:** 1 Neurological Surgery, Montefiore Medical Center, New York, USA; 2 Neurological Surgery, John Hopkins University, Baltimore, USA; 3 Neurological Surgery, Montefiore Medical Center Moses Campus, New York, USA

**Keywords:** alberta stroke program early ct score (aspects), tici, hemorrhagic conversion, mechanical thrombectomy (mt), acute ischemic stroke

## Abstract

Background

Endovascular therapy is known to achieve a high rate of recanalization in patients with acute ischemic stroke (AIS) due to large vessel occlusion (LVO) and is currently the standard of care. Hemorrhagic conversion is a severe complication that may occur following AIS in patients undergoing endovascular thrombectomy (EVT). There is a scarcity of data on the risk factors related to HV in post-EVT patients, especially those who develop symptomatic hemorrhagic conversion. The main objective of our study is to identify independent predictors of radiographic and symptomatic hemorrhagic conversion in our diverse patient population with multiple baseline comorbidities that presented with AIS and were treated with EVT as per the most updated guidelines and practices.

Methodology

This is a retrospective chart review in which we enrolled adult patients treated with EVT for AIS at a comprehensive stroke center in the Bronx, NY, over a four-year period. Bivariate analyses followed by multiple logistic regression modeling were performed to determine the independent predictors of all and symptomatic hemorrhagic conversion.

Results

A total of 326 patients who underwent EVT for AIS were enrolled. Of these, 74 (22.7%) had an HC, while 252 (77.3%) did not. In total, 25 out of the 74 (33.7%) patients were symptomatic. In the logistic regression model, a history of prior ischemic stroke (odds ratio (OR) = 2.197; 95% confidence interval (CI) = 1.062-4.545; p-value = 0.034), Alberta Stroke Program Early CT Score (ASPECTS) of <6 (OR = 2.207; 95% CI = 1.477-7.194; p-value = 0.019), and Thrombolysis in Cerebral Infarction (TICI) 2B-3 recanalization (OR = 2.551; 95% CI = 1.998-6.520; p-value=0.045) were found to be independent predictors of all types of hemorrhagic conversion. The only independent predictor of symptomatic hemorrhagic conversion on multiple logistic regression modeling was an elevated international normalized ratio (INR) (OR = 11.051; 95% CI = 1.866-65.440; p-value = 0.008).

Conclusions

History of prior ischemic stroke, low ASPECTS score, and TICI 2B-3 recanalization are independent predictors of hemorrhagic conversion while an elevated INR is the only independent predictor of symptomatic hemorrhagic conversion in post-thrombectomy patients.

## Introduction

In the United States, over 795,000 people suffer a stroke every year. Of these cases, 87% are categorized as ischemic strokes resulting from a blockage in blood supply to the brain [1]. The pathophysiology of acute ischemic stroke (AIS) includes cardioembolic, large vessel atherosclerotic disease, and small vessel disease, among others [2]. Early complications of AIS include malignant cerebral edema, epileptic seizures, and hemorrhagic transformation [3]. The consequences of AIS contribute to the syndrome being one of the leading causes of death in the United States [1].

Specifically, hemorrhagic conversion has been shown in prior studies to be responsible for long-lasting neurological deficits and an increased risk of mortality [4,5]. Hemorrhagic conversion may be classified into two subtypes, namely, petechial hemorrhage, where the blood accumulates within the confines of the infarcted tissue, and parenchymatous hematoma, where the blood clots and displays a mass effect that may destroy brain tissue [6]. This presents a challenge for neurointerventionalists treating AIS as efforts to recanalize occluded vessels via mainstay anti-thrombotic and thrombolytic treatments, including endovascular thrombectomy (EVT), may paradoxically accelerate parenchymal damage and increase the likelihood of hemorrhagic conversion [7,8]. Although predictors of hemorrhagic conversion in the context of intravenous (IV) thrombolytics have been widely reported, as endovascular thrombectomy is now the standard of care in AIS due to large vessel occlusion (LVO) treatment, more extensive investigation is required regarding its impact on hemorrhagic conversion complication [9].

The purpose of this study is to explore independent predictors of hemorrhagic conversion using a robust, diverse patient population with a high degree of medical comorbidities that underwent EVT as per the current guidelines and evidence-based standards. This investigation is interested in identifying risk factors for all types of hemorrhagic conversion as well as identifying risk factors specifically for symptomatic hemorrhagic conversion. A comprehensive list of variables consisting of patient-specific factors, such as medical comorbidities, presenting vitals, and lab values, along with the clinical presentation, stroke-specific characteristics, and procedural details was included in this analysis. This analysis is valuable as it will allow physicians to anticipate this potential complication of AIS therapy in patients who undergo EVT and offer early intervention brought on by close monitoring post-treatment.

## Materials and methods

This was a retrospective chart review including adult patients who underwent EVT for AIS at our institution in the Bronx, New York, from January 2016 to February 2020. We used electronic medical records (EMRs) to identify eligible patients. Patients were excluded from the study if they were younger than 18 at the time of initial presentation. The date of treatment with EVT at our primary stroke center was set as the index date for further data collection. Using these criteria, 326 patients were included in our study.

For all patients, demographic information was collected, including age, gender, race, and ethnicity. Medical comorbidities pertinent to stroke risk were also collected, which included a history of prior ischemic strokes, coronary artery disease, hypertension, diabetes mellitus, peripheral vascular disease, and smoking status, such as never smoker, current smoker, or former smoker. Previous use of dual antiplatelet therapy (DAPT) was documented.

Important data points pertaining to each patient’s stroke and the care they received prior to EVT were identified, including baseline modified Rankin Scale (mRS), time of symptom onset, time of presentation to the emergency department (ED), hospital transfer status, presenting National Institutes of Health Stroke Scale (NIHSS), Alberta Stroke Program Early CT Score (ASPECTS), and the use of IV tissue plasminogen activator (tPA). The site of occlusion was recorded as either middle cerebral artery (MCA), anterior cerebral artery (ACA), internal carotid artery (ICA), or posterior circulation. Vital signs and lab values at admission were recorded for each patient, which included systolic blood pressure, temperature, white blood cell count, hematocrit, platelet count, blood glucose level, international normalized ratio (INR), and partial thromboplastin time (PTT). Furthermore, we collected information on the thrombectomy procedures for each patient, including procedure length, type of anesthesia used, number of passes, Thrombolysis in Cerebral Infarction (TICI) score, and post-thrombectomy NIHSS. One of our primary outcomes of interest was radiographic evidence of hemorrhagic conversion in patients who underwent EVT on a postoperative computerized tomography (CT) scan prior to discharge. Another primary outcome was symptomatic hemorrhagic conversion which was defined as the occurrence of any new neurological symptom in post-EVT patients who received the diagnosis of radiographic hemorrhagic conversion. Additional outcome measures that were evaluated included length of hospital stay (LOS), discharge disposition, and 90-day mRS scale dichotomized to ≤2 and >2.

Statistical analysis

Bivariate analysis was conducted to compare presenting demographics, medical comorbidities, vital signs and lab values, pre and post-EVT functional neurological assessments, and procedure details of patients without hemorrhagic conversion to patients with any type of hemorrhagic conversion. Bivariate testing was then repeated to compare patients with no hemorrhagic conversion to those who experienced a symptomatic hemorrhagic conversion. Chi-squared tests, Mann-Whitney U tests, and Student’s t-tests were used where appropriate depending on the variable in question. Variables that were found to have a p-value of 0.200 or less on bivariate testing were included in the multiple logistic regression analysis. Multiple logistic regression models were constructed to identify independent predictors of all and symptomatic hemorrhagic conversion. All analyses were conducted using the Stata 16 software (StataCorp., College Station, TX, USA).

## Results

Of the 326 patients included in our study, 74 (22.7%) were found to have hemorrhagic conversion of their infarct on radiographic imaging during their hospital stay. On bivariate analysis of those who had hemorrhagic conversion post-EVT and those who did not, there were significant differences between a history of ischemic stroke, site of occlusion, and ASPECTS score (Table 1). The following variables were included in the multiple logistic regression model for all types of hemorrhagic conversion: gender, prior stroke, diabetes, site of occlusion, temperature, glucose, presenting NIHSS score, ASPECTS score, TICI score, and post-thrombectomy NIHSS. A history of ischemic stroke, low ASPECTS score, and high TICI score were found to be the only independent predictors of hemorrhagic conversion (Table 2).

**Table 1:** Bivariate analysis comparing patients who had a hemorrhagic conversion as a complication of their AIS with those who did not using hypothesis testing. ACA = anterior cerebral artery; ASPECTS = Alberta Stroke Program Early Computed Tomography Score; BP = blood pressure; CAD = coronary artery disease; DAPT = dual antiplatelet therapy; DM = diabetes mellitus; HTN = hypertension; ICA = internal carotid artery; INR = international normalized ratio; IV = intravenous; LOS = length of stay; MCA = middle cerebral artery; PAD = peripheral artery disease; mRS = modified Rankin scale; NIHSS = National Institutes of Health Stroke Scale; PTT = partial thromboplastin time; TICI = Thrombolysis in Cerebral Infarction; tPA = tissue plasminogen activator; WBC = white blood cell count

Characteristics	No hemorrhagic conversion (n = 252)	Hemorrhagic conversion (n = 74)	P-value
Age (year)	68.3 ± 15.4	66.9 ± 14.2	0.505
Gender (male)	115 (45.6%)	43 (58.1%)	0.059
Race			0.211
African American	75 (29.8%)	22 (29.7%)	
Caucasian	49 (19.4%)	21 (28.3%)	
Hispanic	73 (28.9%)	14 (18.9%)	
Other	20 (7.9%)	9 (12.6%)	
Unknown	35 (13.9%)	8 (10.8%)	
Prior stroke	38 (15.1%)	20 (27.0%)	0.018
CAD	38 (15.1%)	11 (14.9%)	0.964
HTN	175 (69.4%)	55 (74.3%)	0.418
DM	80 (31.8%)	31 (41.9%)	0.105
PAD	7 (2.8%)	1 (1.4%)	0.486
Smoker			0.354
Never	166 (67.2%)	46 (63.0%)	
Current	31 (12.6%)	14 (19.2%)	
Former	50 (24.2%)	13 (17.8%)	
Prior DAPT use	13 (5.2%)	5 (6.8%)	0.597
Transfer status (transfer)	156 (61.9%)	42 (57.5%)	0.500
Baseline mRS (0-2)	191 (75.8%)	58 (78.3%)	0.645
Presenting NIHSS (>10)	191 (75.8%)	64 (86.5%)	0.055
Site of occlusion			0.047
MCA	195 (77.7%)	58 (78.4%)	
ICA	23 (9.2%)	13 (17.6%)	
ACA	1 (0.4%)	0 (0%)	
Posterior	32 (12.8%)	3 (4.0%)	
ASPECTS (>6)	243 (96.4%)	67 (90.5%)	0.039
Symptom onset (<6 hours)	172 (68.3%)	53 (71.6%)	0.582
Systolic BP	127 (108-142)	127 (110-142)	0.798
Temperature (°F)	98 (97.5-98.3)	97.9 (97.2-98.2)	0.069
Glucose	127 (106-159)	131.5 (108-194)	0.092
WBC	8.7 (6.6-11.1)	8.5 (7.1-10.7)	0.717
Hematocrit	39.7 (36-43.1)	41.5 (37.6-44)	0.247
Platelet count	226 (186-273.5)	225.5 (179.5-301)	0.928
INR	1 (1-1.2)	1(1-1.2)	0.436
PTT	24.5 (22.6-26.2)	24.4 (23.2-28.4)	0.332
IV tPA	108 (42.9%)	28 (37.8%)	0.441
Procedure length (minutes)	129 (97-155)	122 (97-152)	0.684
Anesthesia (general)	35 (13.9%)	14 (19.2%)	0.266
Number of passes (1)	124 (53.5%)	39 (54.9%)	0.827
TICI (3-2b)	199 (78.9%)	65 (87.8%)	0.087
Post NIHSS (>10)	147 (58.3%)	52 (70.3%)	0.064
Hospital LOS	13.8 ± 6.5	16.2 ± 7.8	0.277
Disposition			0.167
Home	59 (23.4%)	11 (14.9%)	
Acute rehab	106 (42.1%)	30 (40.5%)	
Subacute rehab	9 (3.6%)	3 (4.1%)	
Skilled nursing home	42 (16.7%)	11 (14.9%)	
Died before discharge	36 (14.3%)	19 (25.7%)	
90-day mRS (0-2)	76 (32.9%)	17 (24.6%)	0.193

**Table 2:** Multiple logistic regression model for all types of hemorrhagic conversion seen on radiographic imaging. Ant. Circ. = anterior circulation; ASPECTS = Alberta Stroke Program Early Computed Tomography Score; NIHSS = National Institutes of Health Stroke Scale; TICI = Thrombolysis in Cerebral Infarction

Independent predictor	Adjusted OR (95% CI)	P-value
Gender (male)	1.483 (0.807-2723)	0.204
Prior stroke	2.197 (1.062-4.545)	0.034
Diabetes	1.366 (0.685-2.724)	0.375
Temperature	0.796 (0.586-1.084)	0.143
Glucose	1.000 (0.996-1.005)	0.769
Site of occlusion (Ant. Circ.)	4.043 (0.894-18.286)	0.070
Presenting NIHSS (>10)	0.972 (0.417-2.262)	0.948
ASPECTS (<6)	2.207 (1.477-7.194)	0.019
TICI (2B-3)	2.551 (1.998-6.520)	0.045
Post-NIHSS (>10)	1.792 (0.876-3.664)	0.110

Of the 74 patients who were diagnosed with radiographic hemorrhagic conversion, 25 (33.7%) were symptomatic. Patients who developed symptomatic hemorrhagic conversion were compared to everybody else (Table 3). The only variable that was found to be statistically significant was a post-thrombectomy NIHSS score of greater than 10 (p-value = 0.014). It is important to note that patients who developed symptomatic hemorrhagic conversion of their AIS had worse outcomes than their peers. They had longer average hospital stays and were more often discharged to rehabilitative facilities or died before discharge. Additionally, they had a higher mRS scale at 90 days postoperatively which signifies a worse functional outcome. A multiple logistic regression model was constructed to identify risk factors for symptomatic hemorrhagic conversion specifically (Table 4). The only independent predictor was found to be INR, with a higher INR being associated with a higher odds ratio for symptomatic hemorrhagic conversion.

**Table 3:** Bivariate analysis comparing patients who developed a symptomatic hemorrhagic conversion as a complication of their AIS with those who did not using hypothesis testing. ACA = anterior cerebral artery; ASPECTS = Alberta Stroke Program Early Computed Tomography Score; BP = blood pressure; CAD = coronary artery disease; DAPT = dual antiplatelet therapy; DM = diabetes mellitus; HTN = hypertension; ICA = internal carotid artery; INR = international normalized ratio; IV = intravenous; LOS = length of stay; MCA = middle cerebral artery; PAD = peripheral artery disease; mRS = modified Rankin scale; NIHSS = National Institutes of Health Stroke Scale; PTT = partial thromboplastin time; TICI = Thrombolysis in Cerebral Infarction; tPA = tissue plasminogen activator; WBC = white blood cell count

Characteristics	No symptomatic hemorrhagic conversion (n = 301)	Symptomatic hemorrhagic conversion (n = 25)	P-value
Age (year)	68.3 ± 15.2	64.1 ± 14.8	0.183
Gender (male)	146 (48.5%)	12 (48.0%)	0.961
Race			0.322
African American	90 (29.9%)	7 (28.0%)	
Caucasian	62 (20.6%)	8 (32.0%)	
Hispanic	83 (27.6%)	4 (16.0%)	
Other	25 (8.3%)	4 (16.0%)	
Unknown	41 (13.6%)	2 (8.0%)	
Prior stroke	51 (16.9%)	7 (28.0%)	0.165
CAD	45 (15.0%)	4 (16.0%)	0.888
DM	102 (33.9%)	9 (36.0%)	0.830
HTN	211 (70.1%)	19 (76.0%)	0.534
PAD	7 (2.3%)	1 (4.0%)	0.603
Smoker			0.642
Never	196 (66.4%)	16 (64.0%)	
Current	40 (13.6%)	5 (20.0%)	
Former	59 (20.0%)	4 (16.0%)	
Prior DAPT use	17 (5.7%)	1 (4.0%)	0.729
Transfer status (transfer)	184 (61.3%)	14 (56.0%)	0.600
Baseline mRS (0-2)	228 (75.8%)	21 (84.0%)	0.351
Presenting NIHSS (>10)	233 (77.4%)	22 (88.0%)	0.218
Site of occlusion			0.505
MCA	235 (78.5%)	17 (72.0%)	
ICA	31 (101.3%)	5 (20.00	
ACA	1 (0.3%)	0 (0.0%)	
Posterior	33 (11.0%)	2 (8.0%)	
ASPECTS (>6)	287 (95.4%)	23 (92.0%)	0.456
Symptom Onset (<6 hours)	205 (68.1%)	20 (80.0%)	0.217
Systolic BP	126 (107-140)	134 (125-147)	0.076
Temperature (°F)	98.0 (97.5-98.3)	97.9 (97.3-98.2)	0.197
Glucose	127 (106-166)	135 (109-209)	0.292
WBC	8.7 (6.7-11.1)	7.9 (6.7-9.7)	0.479
Hematocrit	40.0 (36-44)	40.9 (37-44)	0.784
Platelet count	226 (185-279)	214 (170-304)	0.966
INR	1 (1.0-1.2)	1.2 (1.0-2.1)	0.101
PTT	24.4 (22.8-26.2)	25.0 (22.8-29.3)	0.345
IV tPA	124 (41.2%)	12 (48.0%)	0.507
Procedure length (minutes)	125 (97-154)	132 (111-198)	0.315
Anesthesia (general)	43 (14.3%)	6 (25.0%)	0.158
Number of passes (1)	151 (53.7%)	12 (54.5%)	0.942
TICI (3-2b)	245 (81.4%)	19 (76.0%)	0.509
Post NIHSS (>10)	178 (59.1%)	21 (84.0%)	0.014
Hospital LOS	14.1 ± 6.8	17.6 ± 6.7	0.319
Disposition			0.001
Home	68 (22.6%)	2 (8.0%)	
Acute rehab	130 (43.2%)	6 (24.0%)	
Subacute rehab	10 (3.3%)	2 (8.0%)	
Skilled nursing home	51 (16.9%)	2 (8.0%)	
Died before discharge	42 (13.9%)	13 (52.0%)	
90-day mRS (0-2)	90 (32.5%)	3 (13.0%)	0.053

**Table 4:** Multiple logistic regression model for symptomatic hemorrhagic conversion. INR = international normalized ratio; NIHSS = National Institutes of Health Stroke Scale

Independent predictor	Adjusted OR (95% CI)	P-value
Age	0.994 (0.935-1.055)	0.834
Prior stroke	1.923 (0.367-10.069)	0.439
Systolic blood pressure	1.023 (0.984-1.064)	0.244
Temperature	1.001 (0.446-2.247)	0.997
INR	11.051 (1.866-65.440)	0.008
General anesthesia	4.688 (0.683-32.153)	0.116
Post-NIHSS > 10	9.128 (0.515-161.864)	0.132

## Discussion

Hemorrhagic conversion is a known complication of AIS. Hemorrhagic conversion, specifically symptomatic hemorrhagic conversion, has been linked to greater rates of morbidity and mortality in AIS patients [10]. Thus, even though several studies have been published that aim to identify risk factors for hemorrhagic conversion, a general consensus has not been established. The purpose of this study was to answer this question using a large, diverse patient population with a high burden of medical comorbidities that were treated with the current gold standard of AIS treatment, EVT. Our results show that a history of ischemic stroke, a low ASPECTS score, and TICI 2B-3 recanalization are independent predictors for all types of hemorrhagic conversion, while the only independent predictor of symptomatic hemorrhagic conversion is an elevated INR.

Our results show that a history of ischemic stroke, a low ASPECTS score, and near-complete TICI recanalization are independent predictors of all types of hemorrhagic conversion. To understand how each of these factors likely contributes to hemorrhagic conversion, understanding the pathophysiological changes in brain tissue at the cellular level during an acute ischemic injury is vital [11]. When an LVO occurs and perfusion to an area of the brain is decreased for some time, neurons and their supporting cells become irreversibly injured and undergo cell death, which triggers an inflammatory cascade. The cytokines that are released as a result of neuroinflammation act on nearby blood vessels and disrupt the tight junctions between the endothelial cells that comprise the blood-brain barrier. Once the integrity of the blood-brain barrier is compromised, there is an increase in vascular permeability, which is likely what leads to both cytotoxic edema and hemorrhagic transformation. One theory as to why prior ischemic stroke is an independent risk factor for hemorrhagic conversion is, that if an area of brain tissue and surrounding vasculature have previously suffered irreversible ischemic changes and experience a second insult, it will likely be prone to a breakdown which then manifests as an acute bleed. A low ASPECTS score signifies irreversible cellular injury. The risk for bleeding with a low ASPECTS is high given the disintegrating blood-brain barrier in that region [9,12]. The last risk factor for hemorrhagic conversion that our study identified was a high TICI score. The TICI score is used to denote the extent of recanalization achieved by thrombectomy. Typically, the goal of EVT is to achieve complete recanalization of the occluded vessel and total reperfusion to the ischemic cerebral tissue. A TICI score of 3 which signifies full reperfusion has been linked to the restoration of neurological function and better short-term functional outcomes [13]. However, a high TICI score may contribute to hemorrhagic conversion in a multifactorial manner. Because TICI 3 is usually the goal of EVT, some operators can be slightly aggressive in their approach using multiple devices and making more than one attempt to remove the clot. Given the ischemic and inflammatory changes in the target tissue, it is more prone to bleeding, even with minimal trauma. Additionally, once TICI 3 recanalization is achieved, there is a sudden increase in blood flow to the previously ischemic area with a permeable blood-brain barrier which can cause extravasation of blood into the surrounding space. Lastly, the restoration of blood flow to ischemic tissue itself can also cause a secondary reperfusion injury to an already damaged blood-brain barrier.

Another aim of our study was to identify risk factors for symptomatic hemorrhagic conversion. Symptomatic hemorrhagic conversion usually refers to a sizeable intraparenchymal hemorrhage that leads to a deterioration in the patient’s neurological status. It is specifically linked to a worse overall prognosis [10]. The only independent predictor of symptomatic hemorrhagic conversion that our study identified was an elevated INR. The relationship between an elevated INR and symptomatic hemorrhage is self-explanatory. An elevated INR indicates that there is an aberrancy in the coagulation cascade and bleeding is prolonged due to an inability to form an adequate clot. If a patient with an elevated INR develops an intracerebral bleed, there is a strong likelihood that it will be a large volume bleed with a possible mass effect that leads to neurological sequelae. In the literature, an elevated INR has been identified as a risk factor for hemorrhagic conversion in AIS patients, especially those who receive thrombolytic agents [14]. Intravenous thrombolytic agents have been associated with higher rates of hemorrhagic conversion as thrombolytic therapy was the only available treatment option for AIS [15]. It is interesting that IV tPA administration was not found to be associated with hemorrhagic conversion in our analysis. This may be because our study population consisted exclusively of patients who were selected for EVT and, thus, the rates of our patients who received IV tPA may not be reflective of the general AIS population. The underlying mechanism through which IV thrombolytics contribute to hemorrhagic conversion is actively being investigated but is not currently well understood [16]. A study by Lee et al. demonstrated that IV tPA administration can lead to an elevation in INR in a subset of patients in addition to its known effect on fibrinogen levels [17]. Perhaps the missing link is that IV tPA can interfere with the coagulation cascade on a molecular level in some patients, leading to a pro-bleeding state which promotes hemorrhagic transformation. This theory would also explain why not all patients who receive IV thrombolytics experience a hemorrhagic conversion. More research is needed but this possibility cannot be excluded at this time.

A brief review of the literature on the topic of hemorrhagic conversion of AIS is warranted. Prior to the widespread use of EVT to treat AIS, several studies were published to identify predictors for hemorrhagic conversion of AIS. One risk factor was the administration of IV tPA which was the standard treatment for AIS due to LVO at the time [15]. Other risk factors for hemorrhagic conversion included older age, previous stroke, atrial fibrillation, blood thinner use, high presenting NIHSS, elevated systolic blood pressure, hyperglycemia, and low platelet count [18]. Recently, new studies have been published identifying risk factors for hemorrhagic conversion in AIS patients specifically treated with EVT. Some of the risk factors that were identified by these recent studies are the same, such as old age, smoking status, and high presenting NIHSS [19]. Additional variables that have been identified are more thrombectomy specific such as low ASPECTS score, number of passes, use of general anesthesia, increased procedure length, and poor angiographic collateral status [9]. A study by Kaesmacher et al. associated TICI 3 recanalization with a decreased risk for hemorrhagic transformation which is the opposite of our results and illustrates just how divided the literature can be on certain variables [20]. Of note, some studies that were conducted on AIS patients post-EVT also did not show an increased risk for hemorrhagic conversion in patients who received concurrent thrombolytic therapy, which is consistent with our results [19]. Lastly, fewer studies exist in the literature that specifically focus on symptomatic hemorrhagic conversion and they often identify some of the same risk factors as for all radiographic hemorrhagic conversion [21].

There were some inherent limitations to our study. Our study included a relatively modest sample size of slightly over 300 patients. There were only 74 and 25 patients in our two groups of interest. Furthermore, our study design was a retrospective chart review which may be susceptible to biases in data collection and analysis. Additionally, this study did not include specific details on hemorrhagic conversion. We focused on whether the hemorrhagic conversion was symptomatic or not. However, we did not categorize the type of hemorrhagic conversion that occurred for each patient into petechial hemorrhage, or the more deleterious parenchymal hemorrhage. Moreover, important details pertaining to the site or total volume of hemorrhage were not included in our analysis as well as specific details relating to the patients’ symptoms.

## Conclusions

In summary, our results show that a history of prior stroke, a low ASPECTS score, and a high TICI score are associated with radiographic hemorrhagic conversion following AIS in post-EVT patients. Prior ischemic stroke and low ASPECTS score have previously been identified as independent risk factors for hemorrhagic conversion, and our results are in accordance with the current literature. However, our study is unique because it explores the link between near-complete TICI recanalization and increased susceptibility for hemorrhagic conversion. Another strength of our study is that we aimed to identify risk factors for symptomatic hemorrhagic conversion as it has specifically been associated with worse functional outcomes. Our results showed that an elevated INR is an independent predictor of symptomatic hemorrhagic conversion after thrombectomy while IV tPA administration is not. A high TICI score is associated with hemorrhagic conversion prompting continual monitoring of AIS patients who have undergone successful thrombectomy to ensure that they do not develop a serious complication.
